# Performance of a commercially available Generative Pre-trained Transformer (GPT) in describing radiolucent lesions in panoramic radiographs and establishing differential diagnoses

**DOI:** 10.1007/s00784-024-05587-5

**Published:** 2024-03-09

**Authors:** Thaísa Pinheiro Silva, Maria Fernanda Silva Andrade-Bortoletto, Thaís Santos Cerqueira Ocampo, Caio Alencar-Palha, Michael M. Bornstein, Christiano Oliveira-Santos, Matheus L. Oliveira

**Affiliations:** 1 Department of Oral Diagnosis, Division of Oral Radiology, Piracicaba Dental School, University of Campinas, Piracicaba, Sao Paulo, 13414-903 Brazil; 2 Department of Oral Health & Medicine, University Center for Dental Medicine Basel UZB, University of Basel, Basel, 4058 Switzerland; 3 Department of Diagnosis and Oral Health, University of Louisville School of Dentistry, Louisville, KY 40202 USA

**Keywords:** Artificial intelligence, Generative pre-trained transformer, Differential diagnosis, Panoramic radiography

## Abstract

**Objectives:**

To evaluate the performance of a commercially available Generative Pre-trained Transformer (GPT) in describing and establishing differential diagnoses for radiolucent lesions in panoramic radiographs.

**Materials and methods:**

Twenty-eight panoramic radiographs, each containing a single radiolucent lesion, were evaluated in consensus by three examiners and a commercially available ChatGPT-3.5 model. They provided descriptions regarding internal structure (radiodensity, loculation), periphery (margin type, cortication), shape, location (bone, side, region, teeth/structures), and effects on adjacent structures (effect, adjacent structure). Diagnostic impressions related to origin, behavior, and nature were also provided. The GPT program was additionally prompted to provide differential diagnoses. Keywords used by the GPT program were compared to those used by the examiners and scored as 0 (incorrect), 0.5 (partially correct), or 1 (correct). Mean score values and standard deviation were calculated for each description. Performance in establishing differential diagnoses was assessed using Rank-1, -2, and − 3.

**Results:**

Descriptions of margination, affected bone, and origin received the highest scores: 0.93, 0.93, and 0.87, respectively. Shape, region, teeth/structures, effect, affected region, and nature received considerably lower scores ranging from 0.22 to 0.50. Rank-1, -2, and − 3 demonstrated accuracy in 25%, 57.14%, and 67.85% of cases, respectively.

**Conclusion:**

The performance of the GPT program in describing and providing differential diagnoses for radiolucent lesions in panoramic radiographs is variable and at this stage limited in its use for clinical application.

**Clinical relevance:**

Understanding the potential role of GPT systems as an auxiliary tool in image interpretation is imperative to validate their clinical applicability.

**Supplementary information:**

The online version contains supplementary material available at 10.1007/s00784-024-05587-5.

## Introduction

ChatGPT (Chat Generative Pre-trained Transformer), a free service that became available towards the end of 2022, consists of an artificial intelligence (AI) language model with an extensive database that covers various subjects. This AI chatbot has the capability to perform several tasks, such as creating spreadsheets for different types of data, generating analogies, outlining research topics, and providing study notes based on a given topic. In addition to those interesting functionalities, this tool has the ability to generate image descriptions from hyperlinks when provided with specific prompts, which holds potential significance in the field of dentomaxillofacial radiology (DMFR) [1]. To date, the scientific literature includes numerous review papers and editorial letters, yet a notable gap exists in the form of empirical studies assessing the performance of a commercially available tool such as ChatGPT [2].

Image analysis and description are among the primary responsibilities of radiologists, carrying significant indirect impact on patients’ treatments [3]. Radiologists face numerous challenges in interpreting medical images. The complexity and variability of imaging modalities in DMFR encompass a wide range of two and three-dimensional images, including periapical radiography, panoramic views, and cone-beam computed tomography. Each modality presents unique challenges in analysis and diagnosis [4]. Additionally, the volume of imaging examinations has significantly increased over time, leading to a heavier workload. Subjective interpretation and variability in image descriptions can hinder effective communication among healthcare professionals, potentially compromising patient care [5–7].

Panoramic radiography is a two-dimensional image modality that enables visualization of the teeth in the upper and lower jaws, and the surrounding structures of the maxillofacial complex. By providing broad views at a relatively low radiation dose and relatively low cost, panoramic radiography is widely available and often used for the evaluation, diagnosis, and monitoring of jaw lesions [8]. Radiolucent lesions are the most prevalent type, and interpreting and describing them can be challenging, either due to the non-specific clinical presentation or the inherent limitations of this imaging modality, such as image blurring, distortion, and superimposition of structures [9–14].

Panoramic radiography is a commonly employed imaging technique to evaluate lesions within the maxillofacial complex. Additionally, acknowledging the potential application of commercially available GPT programs as auxiliary instruments in image analysis – encompassing the provision of supplementary insights, generation of descriptive narratives, and presentation of differential diagnose – emphasizes the importance of their role. Consequently, it is crucial for GPT programs to demonstrate accuracy to fulfill their intended functions for image diagnosis in dental medicine effectively.

## Materials and methods

### Sample selection

A search for panoramic radiographs was conducted on the open-access websites Radiopaedia.org (https://radiopaedia.org/) and CDI Peru (https://cdi.com.pe/), which provide well-documented clinical cases. The inclusion criteria were clinical cases with panoramic radiographs displaying permanent dentition and a single radiolucent lesion in the maxilla or mandible, with histopathological confirmation or a probable differential diagnosis, and presenting specific hyperlinks that redirected to the corresponding isolated panoramic radiograph (i.e., not the full webpage with textual information). Blurry, cropped, or edited panoramic radiographs containing annotations such as arrows, circles, lines, or texts were not included in this study. This resulted in a total of 28 panoramic radiographs included for further analysis (Annex 1).

### Reference standard description and diagnostic impressions

**Fig. 1:**
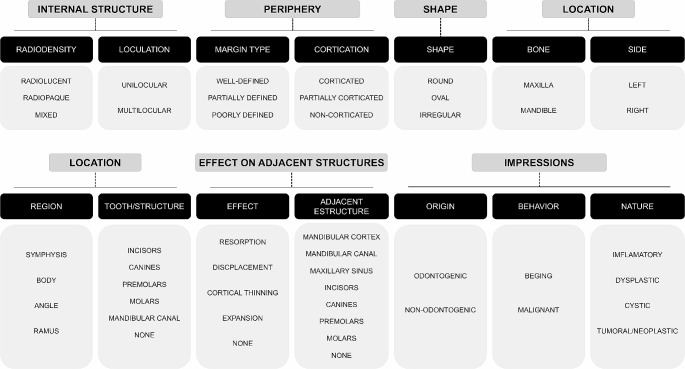
Predetermined topics, parameters and keywords addressed by the examiners to describe the lesions in all panoramic radiographs

Three experienced oral and maxillofacial radiologists evaluated all panoramic radiographs in consensus and described the radiographic aspects of the lesions according to the following parameters: internal structure (radiodensity and loculation), periphery (margin type and cortication), shape, location (bone, side, region, and tooth/structure), and effect on adjacent structures (effect and adjacent structure). Then, based on the descriptions, the examiners determined their impressions, as follows: origin (odontogenic or non-odontogenic), behavior (benign or malignant), and nature (inflammatory, dysplastic, cystic, or tumoral/neoplastic). Figure 1 depicts the predetermined standardized topics and parameters used in the descriptions, along with the corresponding keywords employed by the examiners to describe the lesions in all panoramic radiographs.

### Description based on the commercially available GPT program

Using the free online ChatGPT program (GPT-3.5 model) available at https://chat.openai.com, three prompts were submitted accompanied by the hyperlink of each individual panoramic radiograph: (1) Description: “Please describe the radiographic aspects of the lesion in this panoramic radiograph according to the following topics: internal structure, unilocular or multilocular, periphery, shape, location, and effect on adjacent structures.“, (2) Impressions: “Based on the previous radiographic aspects, what are your impressions of the lesion regarding its origin, behavior, and nature?“, and (3) Differential diagnoses: “What are the likely differential diagnoses, in order of priority, based on the radiographic features noted in the previous panoramic radiograph?”. All output responses generated by the GPT program were carefully reviewed by the same three experienced oral and maxillofacial radiologists in consensus, who identified and extracted the keywords employed by the program to describe the lesions.

### Description performance scoring of the commercially available GPT

**Fig. 2:**
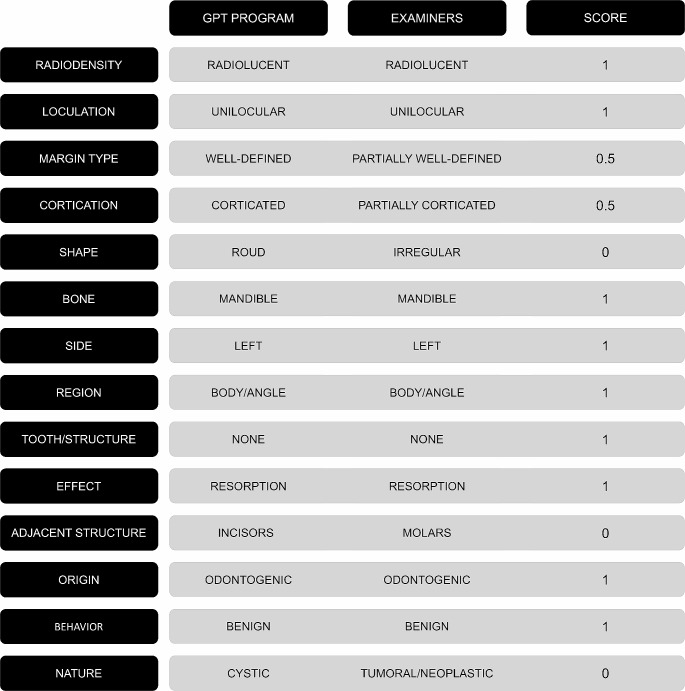
Example of application of the scoring method for evaluating the GPT program’s performance in describing a representative radiolucent lesion. 1, correct; 0.5, partially correct; 0 incorrect

The performance of the GPT program in describing the lesions in the panoramic radiographs was scored by confronting the extracted keywords with those determined by the reference standard established by the radiologists. When the keywords for each parameter employed by the GPT program matched those from the reference standard, the description was considered correct and a score of 1 was assigned. When the keyword employed by the GPT program was similar but not identical to the reference standard, the description was considered partially correct and a score of 0.5 was assigned. Finally, when the keyword employed by the GPT program was different or when more than one keyword was described for the same lesion, with one being correct and the other(s) incorrect, the description was considered to be incorrect and a score of 0 was assigned. Figure 2 illustrates the application of the scoring method for evaluating the GPT program’s performance in describing a representative radiolucent lesion.

Means and standard deviations were calculated from the scores obtained for each addressed parameter of all panoramic radiographs. The scores were also divided into two groups based on the extension of the lesion (spanning one or two/three regions) and presented in the form of a bar chart.

### GPT program’s rank-n differential diagnoses

Rank-1, Rank-2, and Rank-3 were manually computed to evaluate the performance of the GPT program’s differential diagnosis in comparison to the confirmed or probable diagnoses of each case. Rank-n accuracy is defined as the percentage of finding the n^th^ matching target [15, 16]. For instance, Rank-3 accuracy is the percentage of cases in which the correct diagnosis was included in the top three most likely differential diagnoses.

## Results

**Fig. 3:**
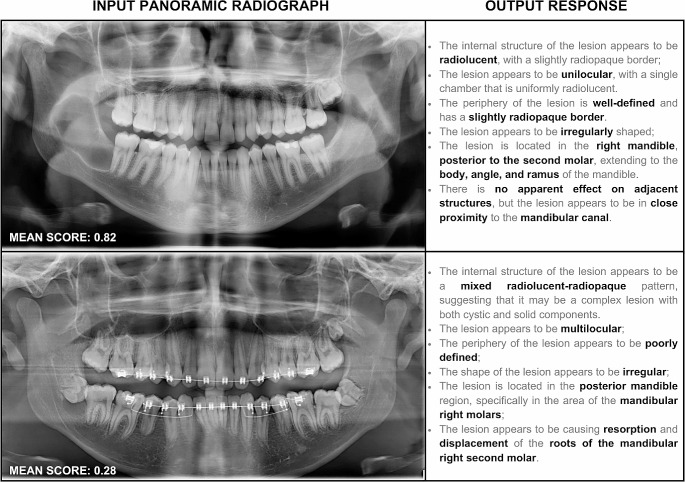
Representative panoramic radiographs and their corresponding divergent outputs from the GPT program, leading to a higher score (mostly correct output) in the top image and a lower score (mostly incorrect output) in the bottom image

The GPT program’s description performance varied considerably among each parameter assessed. The highest mean score values (i.e., above 0.80) were 0.93 for margin type and affected bone, and 0.87 for origin. These parameters also demonstrated to be more consistently described by the GPT program, as evidenced by their relatively lower standard deviation values. Conversely, shape, region, teeth/structures, effect, affected region, and nature showed considerably lower mean score values, ranging from 0.22 to 0.50, along with higher standard deviation values, reflecting greater variability in the response pattern. Finally, the parameters radiodensity, loculation, cortication, side, and behavior presented intermediate performance with mean score values ranging from 0.61 to 0.77 and also revealing a highly variable standard deviation (Table 1). The overall performance of the GPT program in describing radiolucent lesions in panoramic radiographs, with all parameters considered, revealed a mean score value of 0.56, and a standard deviation of 0.48. Figure 3 illustrates two cases where the GPT program exhibited divergent performance (output mostly correct vs. incorrect).

**Table 1:** Mean score values and standard deviation (SD) of the GPT program’s performance in describing radiolucent lesions in panoramic radiographs, as a function of the assessed topic and parameter

	Topic	Parameter	Mean	SD
Description	Internal Structure	Radiodensity	0.75	0.44
		Loculation	0.61	0.50
	Periphery	Margin type	0.93	0.22
		Cortication	0.73	0.29
	Shape	Shape	0.43	0.50
	Location	Affected bone	0.93	0.26
		Side	0.68	0.48
		Region	0.34	0.39
		Teeth/Structures	0.32	0.46
	Effect on Adjacent Structures	Effect	0.32	0.47
		Affected region	0.22	0.39
Impressions	Origin		0.87	0.35
	Behavior		0.77	0.43
	Nature		0.50	0.49
Total			0.56	0.48

**Fig. 4:**
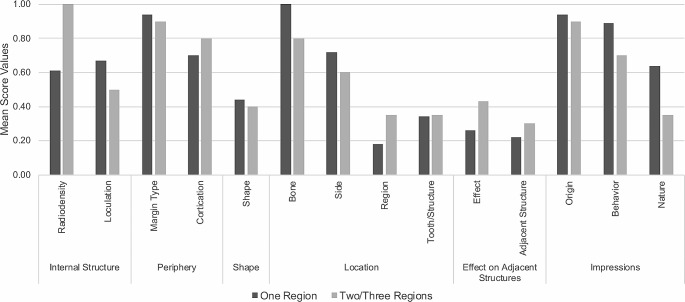
Mean score values of the GPT program’s performance in describing radiolucent lesions in panoramic radiographs, as a function of the assessed topic and parameter and the extension of the lesion (one or two/three regions)

The performance of the GPT program in describing radiolucent lesions in panoramic radiographs did not exhibit a consistent pattern when the lesion spanned one or two/three regions. Overall, slight differences in description performance were observed in the following parameters: cortication, shape, teeth/structures, affected region and origin. However, it was not possible to establish a pattern of higher performance based on the extension of the lesion. Radiodensity, loculation, margin type, bone, side, region, effect, behavior, and nature showed the largest differences in performance considering the extension of the lesion, with lesions spanning one region generally being more accurately described than those spanning two/three regions (Fig. 4).

Regarding the GPT program’s performance in establishing differential diagnoses, as shown in Table 2, seven cases (25%) were accurately predicted as the first diagnostic hypothesis, while 16 cases (57.14%) and 19 cases (67.85%) were accurately predicted within the first two and the first three diagnostic hypotheses, respectively. In nine cases (32.14%), the GPT program did not succeed in accurately predicting them within the three diagnostic hypotheses.

**Table 2:** Absolute number and relative percentage of cases of the GPT program’s performance in predicting rank-1, -2, -3 differential diagnoses, and cases in which no correct differential diagnosis was established

Rank-n	Prediction Performance	
	Number of cases	Percentage of cases
Rank-1	7	25.00
Rank-2	16	57.14
Rank-3	19	67.86
None	9	32.14

## Discussion

ChatGPT is a recently launched powerful chatbot that has the potential to answer questions and respond to commands in an impressively short time with well-structured and well-written sentences, which can strongly convince or persuade readers of its accuracy and reasoning. Among a large list of capabilities, this openly available GPT program describes radiographic lesions in a coherent structure when provided with a hyperlink to a radiographic image and specifically asked to do so. Within the methodological design and limitations of the present study, the GPT program showed a limited and highly variable performance in describing and establishing differential diagnoses for radiolucent lesions in panoramic radiographs. Such variability raises questions about the GPT program’s reliability and suitability for clinical application at this time.

Specificity was a relevant aspect that had to be taken into consideration during the development of the three prompts for the present study, as observed in pilot studies (data not shown), due to limitations in the studied GPT program. The prompts had to clearly specify the imaging modality and acknowledge the presence of a lesion, otherwise, the GPT program would generate responses focused on explaining the imaging modality, limitations, and indications, rather than providing an actual description of the lesion of interest in the image. This reveals that the responses generated by this GPT program are strongly prompt-dependent. Importantly, the present study focused on a customary clinical scenario when a radiologist or a clinician asks for the opinion of colleagues when spotting a lesion. If the intention was to evaluate the ability of the GPT program to spot the lesion, then the prompt would have to be specific for that purpose.

The studied GPT program provided incomplete and unspecific information in many cases, and this was negatively considered in the scoring system for the description performance. There were instances when an effect on the adjacent structure, such as cortical thinning, was mentioned without indicating the location. In some other circumstances, cortical thinning was described as affecting the buccal or lingual plates, despite the two-dimensional nature of panoramic radiography, which hinders the proper visualization of these structures. Such inconsistencies certainly led to variable and inconsistent results seen in the present investigation. Approximately 46% of the responses exhibited contradictions. For example, some lesions were described as both “corticated” and “aggressive due to its diffuse borders”. This behavior raises the hypothesis that the GPT program used may not perform a detailed evaluation of the image. Instead, it may rely on general patterns and commonly available information regarding radiolucent lesions in panoramic radiographs.

Surprisingly, some cases were very well described, pointing to highly specific anatomical references, such as the cement-enamel junction. One hypothesis for the success in describing such cases is the potential redirection from the hyperlink to the online platform from which the case was taken, or the potential extraction of the identification of the lesion from the hyperlink. This might have led this GPT program to use more common characteristics associated with that specific lesion. Another hypothesis for the accurate descriptions is that possibly the GPT program works based on prevalence. Considering that the sample in this study consisted of benign radiolucent lesions, characteristics like density, margination, and affected bone are more predictable, increasing the chances of a good performance by guessing the most common presentation. However, for other characteristics that exhibit greater variation from one lesion to another, such as the affected region, relationship with teeth or structures, effects, and the location of those effects, prevalence-based assumptions become more challenging. As a result, lower mean score values with a wider standard deviation were observed for these characteristics.

Irrespective of the responses generated by the GPT program used, it is worth mentioning that all of them ended with a sentence emphasizing the importance of a radiologist providing the final description and confirming the diagnosis of the lesion through histopathological examination. Despite the nicely structured and apparently convincing responses, this highlights that the present GPT program recognizes its limited reliability when generating responses or handling open questions, and stresses that the responsibility for verifying and confirming the appropriate course of action based on the information provided still lies with the radiologist.

Regarding the present GPT program’s performance in establishing differential diagnoses, it’s important to highlight that it can achieve a high accuracy solely based on the prevalence reported in the literature, without the need to interpret the actual image. Furthermore, since GPT is an artificial intelligence-based tool, it is in a constant process of learning and evolution [1]. Therefore, it is expected to have the potential for improvement, particularly in enhancing its performance in the aspects studied in the current research.

The advent of AI, demonstrated by the remarkable capabilities of GPT programs like ChatGPT and ClinicGPT [17], has introduced transformative possibilities and challenges to the field of DMFR. As part of our community may struggle with the integration of AI, it is evident that this technology holds huge potential for reshaping both education and practice [18]. Nevertheless, this evolution comes with an inherent set of challenges. Many radiologists, traditionally untrained in GPT-based practice, must dedicate themselves to assess this application effectively. Furthermore, ethical and legal considerations are significant, demanding a collective consensus for the responsible implementation of GPT programs in DMFR.

The scientific literature has consistently indicated strong performance when using deep learning-based AI technologies for tasks encompassing the reading and interpretation of radiographic images in dental medicine. Examples include the assessment of root morphology of the mandibular first molar, automatic classification of odontogenic keratocysts and ameloblastomas, differential diagnosis of lingual mandibular bone depression (e.g. Stafne defect) from true pathological radiolucent cysts or tumors, and classification and localization of odontogenic lesions [19–22].

It is imperative to recognize that, unlike convolutional neural networks (CNNs), ChatGPT primarily functions as a language model, and while its capabilities have been extended to include image handling, it may not be as specialized as traditional image-centric models. In the domain of oral radiology, traditional research often leverages CNNs for image analysis, which typically involves specialized image-processing models adept at detecting patterns and features within radiographic images. In contrast, ChatGPT employs a distinctive approach with the possibility of generating natural language descriptions related to these images. It has the potential to furnish contextual information, explanations, or summaries based on visual content [1], presenting a complementary perspective to the image-focused methodologies, which are commonly used in the field of oral radiology. Furthermore, previous studies have suggested that ChatGPT-3, -3.5 and − 4 can accurately establish differential diagnosis, which contrasts with the results highlighted in the present study [23, 24]. This discrepancy may be attributed to the fact that the prompts employed in those studies utilizing ChatGPT comprised short clinical textual descriptions, in contrast to the methodology adopted in the present investigation, which utilized hyperlinks redirecting to specific panoramic images. Evidently, the lack of transparency regarding the training data and methodologies utilized in ChatGPT’s development poses challenges in fully understanding its capabilities and potential biases. Furthermore, the absence of a dedicated training process using task-specific medical image data is a relevant limitation of the present investigation that merits consideration.

The deployment of external AI tools as diagnostic aids necessitates careful consideration of data privacy and security concerns. Due to potential risks associated with uploading sensitive data, such as patient images, to public servers, the need for robust protective measures has to be considered. In alignment with these concerns, we emphasize the importance of implementing stringent security protocols when integrating AI technologies into clinical applications. While our current study utilized publicly available panoramic radiographs, we acknowledge that real-world clinical scenarios demand heightened attention to privacy and accountability, aligning with national requirements for medical patient data protection laws.

To the best of the author’s knowledge, the present study is the first to evaluate the performance of a commercially available GPT program in describing and establishing differential diagnoses of lesions based on radiographic images. This limitation severely restricts the possibility of making direct comparisons with previous studies. Furthermore, the current research study evaluated panoramic radiographs from freely available clinical data repositories on online platforms, focusing specifically on the presence of radiolucent lesions. Our sample size was limited by our strict inclusion criteria, which was necessary to mitigate potential bias from hyperlinks to images embedded on a website containing all textual information and graphic annotations. Further studies utilizing images from the same databases, following the same quality control procedures, and including radiopaque and mixed lesions are encouraged for a more comprehensive analysis. In addition, considering that the GPT program model used when this study was conceived only allowed reading images through their hyperlinks (GPT-3.5 model), future research assessing the performance of the latest update of this transformer dating back to September 2023 are encouraged. This latest program model allows for image uploads directly to the system. This is important because the method through which AI-based language models extract data may differ. Furthermore, this also sheds light on the inherent limitation of studies assessing rapidly evolving systems, as the one assessed herein, as evidence-based conclusions are more likely exposed to the risk of becoming outdated shortly. Conversely, the significance of such studies relies on the possibility of reliably tracking their technological evolution process.

## Conclusion

Up to the point in time this study was conducted, the performance of the ChatGPT-3.5 model in describing radiolucent lesions in panoramic radiographs and establishing differential diagnoses is variable and limited and is unsuitable for clinical application.

### Electronic supplementary material

Below is the link to the electronic supplementary material.

Supplementary Material 1

## Data Availability

No datasets were generated or analysed during the current study.

## References

[1] ChatGPT (2023) : Optimizing Language Models for Dialogue. https://openai.com/blog/chatgpt/ (accessed 30

[2] Ocampo TSC, Silva TP, Alencar-Palha C, Haiter-Neto F, Oliveira ML (2023). ChatGPT and scientific writing: a reflection on the ethical boundaries. Imaging Sci Dent.

[3] European Society of Radiology (ESR) (2022) The role of radiologist in the changing world of healthcare: a White Paper of the European Society of Radiology (ESR). Insights Imaging 13 :100. Published 2022 Jun 4. 10.1186/s13244-022-01241-4 10.1186/s13244-022-01241-4 PMC9167391 35662384

[4] White SC, Pharoah MJ (2015). Radiologia oral: Princípios E Interpretação.

[5] Ruutiainen AT, Durand DJ, Scanlon MH, Itri JN (2013). Increased error rates in preliminary reports issued by radiology residents working more than 10 consecutive hours overnight. AcadRadiol.

[6] Hanna TN, Shekhani H, Lamoureux C (2017). Emergency radiology practice patterns: shifts, schedules, and job satisfaction. J Am Coll Radiol.

[7] Patlas MN, Katz DS, Scaglione S (2019). Errors in emergency and trauma radiology.

[8] Różyło-Kalinowska I (2021). Panoramic radiography in dentistry. Clin Dent Rev.

[9] Koivisto T, Bowles WR, Rohrer M (2012). Frequency and distribution of radiolucent jaw lesions: a retrospective analysis of 9,723 cases. J Endod.

[10] Flint DJ, Paunovich E, Moore WS, Wofford DT, Hermesch CBA (1998). Diagnostic comparison of panoramic and intraoral radiographs. Oral Surg Oral Med Oral Pathol Oral Radiol Endod.

[11] Akkaya N, Kansu O, Kansu H, Cagirankaya LB, Arslan U (2006). Comparing the accuracy of panoramic and intraoral radiography in the diagnosis of proximal caries. Dentomaxillofac Radiol.

[12] Akarslan ZZ, Akdevelioğlu M, Güngör K, Erten H (2008). A comparison of the diagnostic accuracy of bitewing, periapical, unfiltered and filtered digital panoramic images for approximal caries detection in posterior teeth. Dentomaxillofac Radiol.

[13] Dunfee BL, Sakai O, Pistey R, Gohel A (2006). Radiologic and pathologic characteristics of benign and malignant lesions of the mandible. Radiographics.

[14] Devenney-Cakir B, Subramaniam RM, Reddy SM, Imsande H, Gohel A, Sakai O (2011). Cystic and cystic-appearing lesions of the mandible: review. AJR Am J Roentgenol.

[15] Fan F, Ke W, Wu W, Tian X, Lyu T, Liu Y (2020). Automatic human identification from panoramic dental radiographs using the convolutional neural network. Forensic Sci Int.

[16] Simonyan K, Zisserman A Very deep convolutional networks for large-scale image recognition. arXiv preprint arXiv:1409.1556 2014

[17] Zhou KX (2023) Introducing ClinicGPT: A custom large language model for institutional dental clinics. *J Dent Educ*. Aug 4. doi: 10.1002/jdd.13348. Epub ahead of print. PMID: 37539925 10.1002/jdd.13348 PMC11675521 37539925

[18] Islam NM, Laughter L, Sadid-Zadeh R, Smith C, Dolan TA, Crain G, Squarize CH (2022) Adopting artificial intelligence in dental education: A model for academic leadership and innovation. *J Dent Educ*. ; 86:1545–1551. doi: 10.1002/jdd.13010. Epub 2022 Jul 3. PMID: 35781809 10.1002/jdd.13010 35781809

[19] Hiraiwa T, Ariji Y, Fukuda M, Kise Y, Nakata K, Katsumata A, Fujita H, Ariji E (2019). A deep-learning artificial intelligence system for assessment of root morphology of the mandibular first molar on panoramic radiography. Dentomaxillofac Radiol.

[20] Bispo MS, Pierre Júnior MLGQ, Apolinário AL, Santos D, Junior JN, Neves BC, Crusoé-Rebello FS (2021). Computer tomographic differential diagnosis of ameloblastoma and odontogenic keratocyst: classification using a convolutional neural network. Dentomaxillofac Radiol.

[21] Ha EG, Jeon KJ, Lee C, Kim HS, Han SS (2023). Development of deep learning model and evaluation in real clinical practice of lingual mandibular bone depression (Stafne cyst) on panoramic radiographs. Dentomaxillofac Radiol.

[22] Kang J, Le VNT, Lee DW, Kim S (2024). Diagnosing oral and maxillofacial diseases using deep learning. Sci Rep.

[23] Hirosawa T, Harada Y, Yokose M, Sakamoto T, Kawamura R, Shimizu T (2023). Diagnostic accuracy of Differential-diagnosis lists generated by Generative Pretrained Transformer 3 Chatbot for Clinical vignettes with Common Chief complaints: a pilot study. Int J Environ Res Public Health.

[24] Hirosawa T, Kawamura R, Harada Y, Mizuta K, Tokumasu K, Kaji Y, Suzuki T, Shimizu T (2023). ChatGPT-Generated Differential diagnosis lists for Complex Case-Derived Clinical vignettes: diagnostic accuracy evaluation. JMIR Med Inf.

