# Supplementary material for: Performance of a commercially available Generative Pre-trained Transformer (GPT) in describing radiolucent lesions in panoramic radiographs and establishing differential diagnoses
Source: Clin Oral Investig. 2024 Mar 9;28(3):204. doi: 10.1007/s00784-024-05587-5 (PMC10924032; doi:10.1007/s00784-024-05587-5)
Supplement: Supplementary file 1 — Supplementary Material 1 [file 784_2024_5587_MOESM1_ESM.pdf]

Annex 1: Diagnosis and hyperlinks of the 28 selected panoramic radiographs

**1**	Ameloblastoma	https://prod-images-static.radiopaedia.org/images/10560960/f34e52a3f23562759a0fa6f8b3e868_jumbo.jpeg
**2**	Ameloblastoma	https://prod-images-static.radiopaedia.org/images/30262258/de2f65b95c11609cb9b0198d78d9af_jumbo.jpeg
**3**	Ameloblastoma	https://d2jx2rerrg6sh3.cloudfront.net/image-handler/ts/20211117122227/ri/673/picture/2021/11/shutterstock_1084595309.jpg
**4**	Ameloblastoma	https://media.springernature.com/full/springer-static/image/art%3A10.1186%2Fs13256-016-1025-1/MediaObjects/13256_2016_1025_Fig1_HTML.gif?as=webp
**5**	Ameloblastoma	https://cdi.com.pe/wp-content/uploads/2011/08/468.png
**6**	Calcifying Epithelial Odontogenic Tumor	https://cdi.com.pe/wp-content/uploads/2009/02/Caso-No-336-TUMOR-ODONTOGENICO-EPITELIAL-CALCIFICANTE-TOEC.png
**7**	Dentigerous Cyst	https://prod-images-static.radiopaedia.org/images/20855196/59f0ba68941384f4108b9c8d715f14_jumbo.jpeg
**8**	Dentigerous Cyst	https://prod-images-static.radiopaedia.org/images/23748986/b5e8814b7bd13806dcab9c26ba030b_jumbo.jpeg
**9**	Dentigerous Cyst	https://prod-images-static.radiopaedia.org/images/20855196/59f0ba68941384f4108b9c8d715f14_jumbo.jpeg
**10**	Dentigerous Cyst	http://userscontent2.emaze.com/images/ec4ac35b-aefb-4427-a0ad-34186f0aa152/41cd6181-406b-40f3-b0bc-3b9352e20860.jpg
**11**	Dentigerous Cyst	https://cdi.com.pe/wp-content/uploads/2020/08/Figura-1-PANORMAICA.jpg
**12**	Dentigerous Cyst	https://prod-images-static.radiopaedia.org/images/52596306/e736fa1af6c9dce203afcde9ee68e61b5ea8ec01ffad4e525be0d1a3082067aa_jumbo.jpeg
**13**	Dentigerous Cyst	https://cdi.com.pe/wp-content/uploads/2020/11/1-1-1024x499.jpg
**14**	Lateral Periodontal Cyst	https://case.edu/dental/sites/case.edu.dental/files/2018-04/Lateral_Periodontal_Cyst.jpg
**15**	Odontogenic Glandular Cyst	https://prod-images-static.radiopaedia.org/images/6583976/bc15f98468c40f9e07116a43bc22f1_jumbo.jpg
**16**	Odontogenic Keratocyst	https://prod-images-static.radiopaedia.org/images/34598265/75b004c31bf920e6063162a10ae1b8_jumbo.jpeg
**17**	Odontogenic Keratocyst	https://prod-images-static.radiopaedia.org/images/17618813/e9db75a9e87c79814eeba23f8c300e_jumbo.jpeg
**18**	Odontogenic Keratocyst	https://prod-images-static.radiopaedia.org/images/10558319/75a52488c2efd70e5b21916d9f4cb3_jumbo.jpeg
**19**	Odontogenic Keratocyst	https://cdi.com.pe/wp-content/uploads/2022/05/pano-2.jpg
**20**	Odontogenic Keratocyst	https://prod-images-static.radiopaedia.org/images/18311870/94f4e6d41b02410277102489007684_jumbo.jpeg
**21**	Odontogenic Keratocyst	https://cdi.com.pe/wp-content/uploads/2021/03/PANO.jpg
**22**	Odontogenic Keratocyst	https://cdi.com.pe/wp-content/uploads/2023/05/PANO.png
**23**	Odontogenic Keratocyst	https://cdi.com.pe/wp-content/uploads/2019/11/Diapositiva2.jpg
**24**	Odontogenic Keratocyst	https://cdi.com.pe/wp-content/uploads/2019/10/CASO-608-QUISTE-ODON-TOGENICO-IMG-1.png
**25**	Odontogenic Myxoma	https://cdi.com.pe/wp-content/uploads/2018/07/CASO-609-MIXOMA-IMG-1-1024x499-1.jpg
**26**	Paradental Cyst	https://prod-images-static.radiopaedia.org/images/61438760/f09e808b6e2f9a7452aa29bc2d4afa9c81e3999a6ab712cbc771e0b42d79361f_jumbo.jpeg
**27**	Radicular Cyst	https://prod-images-static.radiopaedia.org/images/55443180/periapical_cyst_correct_jumbo.jpeg
**28**	Stafne's Bone Defect	https://cdi.com.pe/wp-content/uploads/2014/01/510.png
